# Chia (*Salvia hispanica*) Seed Oil Modulates the Haemato-Immunological Response, Antioxidative Status and Cytokine Gene Expression of Tropical Freshwater Teleost, *Labeo rohita*

**DOI:** 10.3390/biology14010095

**Published:** 2025-01-18

**Authors:** Sanjay Kumar Gupta, Rajan Gupta, Akruti Gupta, Md Javed Foysal, Kishore Kumar Krishnani

**Affiliations:** 1 ICAR-Indian Institute of Agricultural Biotechnology, Ranchi 834010, India; 2 Department of Biotechnology, Vinoba Bhave University, Hazaribag 825301, India; 3 School of Molecular and Life Sciences, Curtin University, Bentley, WA 6102, Australia; 4 School of Environmental and Life Sciences, The University of Newcastle, Callaghan, NSW 2308, Australia; 5 Department of Genetic Engineering and Biotechnology, Shahjalal University of Science and Technology, Sylhet 3114, Bangladesh

**Keywords:** chia seed oil, haemato-immunology, enzyme assay, cytokine gene, *L. rohita*

## Abstract

The present investigation attempts to evaluate the impact of the dietary inclusion of chia (*Salvia hispanica*) seed oil (CSO) on the indices of haemato-immunology, metabolic enzymes, and expression of immune-responsive cytokine genes in *Labeo rohita* (rohu) fingerlings. The responses were observed in a 60-day feeding trial, set up with a total of 180 rohu fingerlings (19.74 ± 0.33 g) randomly allocated to four treatment groups with three replicates each. The groups were fed with a basal diet incorporated with 0%, 1.0%, 2.0%, and 3.0% CSO, denoted as control, CSO (1), CSO (2), and CSO (3), respectively. Significant (*p* < 0.05) augmentation of hematological indices such as total protein and globulin levels was observed in the group fed a 1.0% CSO-supplemented diet. Serum glucose, cholesterol, triglycerides, and complement reactive protein levels declined, whereas marker anti-oxidative enzymes (SOD, CAT, and GST) and protein metabolic enzymes (ALT and AST) increased (*p* < 0.05) in the lowest CSO-supplemented group. A significant upregulation of inflammatory cytokine viz. IL-1β, IFN-γ, TNF-α, and TLR22 alongside downregulation of IL-10 was noted in various tissues. The results support the inclusion of 1.0% CSO as a prospective dietary vegan substitute to fish oil in rohu aquaculture.

## Simple Summary

Chia seed oil is known as a powerhouse source of omega-3 (ω3) fatty acids, is rich in therapeutic bioactive components and natural antioxidants, and shows antimicrobial, anti-inflammatory, and heath-promoting properties in animals. The current research portrays the advantages of chia seed oil supplementation to address the unsustainable usage of fish oil in aquafeeds as well as to promote immunonutrition in tropical freshwater fish. The physiological and molecular responses tested through various endpoints depicted enhanced performance at the lowest level of CSO supplementation. However, higher dosages of dietary CSO supplementation at 2% and 3% led to a remarkable decline in haemato-immunological and biochemical responses. The results of the current study suggest the supplementation of chia seed oil at 1% to potentially improve the health performance and immunity of *L. rohita* fingerlings, whereas higher levels of CSO incorporation induced metabolic stress and immuno-suppression.

## 1. Introduction

Aquaculture across the world has grown exponentially, and production in the past 3 decades has increased by 1200% [1,2]. Fish oil incorporation is a major component in compound-feed formulation, accounting for a 60% utilization share as a lipid source in the aquaculture sector at a global level [3]. It has been proven to augment the health and immunity of cultivable fishes across the aquaculture feed industry [4]. However, due to over exploitation and the consequent severe decline in the catch of food-grade marine fish resources, the search for plant-based alternatives and sustainable lipid sources that can provide comparable nutritive value to fish oil has intensified in the past few years [5].

*Labeo rohita* (rohu) ranks as the eleventh top species among all global aquaculture species [1]. India holds its predominant second position in global aquaculture, with a contribution of over 75% in the production of Indian major carps, including *L. rohita* [6]. The species is widely cultured as the most abundant Indian Major Carp (IMC) in India, with an annual production of 4.407 million tons. To meet the sustainable demand of this extensively cultured carp species, multiple vegan options are currently being sought. The replacement of fish oils with different vegetable oils, such as soybean oil, sunflower oil, linseed oil, flaxseed oil, rapeseed oil, canola oil, chamomile oil, palm oil and olive oil, has been tried in the past, with varying degrees of success [7]. However, the recent inclusion of chia seed oil (CSO) in the diets of freshwater and marine fish species has shown promising prospects and is mainly substituted as a cheaper and sustainable replacement of fish oil, attributed to its feasibility, price stability, and quality [8].

The plant *Salvia hispanica* (Chia) is an annual herbaceous oilseed member of the family Lamiaceae [9]. The plant has thus been widely known for its seeds that contain approximately 25–39% oil by weight [10]. The oil derived from chia seeds is regarded as a well-known vegetable oil rich in omega-3 (ω3) fatty acids, proteins, fibers, vitamins, minerals, and natural antioxidants [11]. It is also an abundant source of n-3 C-18 polyunsaturated fatty acids (PUFA), namely linoleic acid, LA (2n-6; 17–26%), α-linolenic acid, and ALA (3n-3, 50–57%), crucial for health, antioxidant, and antimicrobial activity [12]. In fact, CSO is reported to possess a higher n-3 PUFA content than flax, soyabean, or canola seed oils [13]. A large number of therapeutic bioactive components including tocopherols, polyphenols, carotenoids, phospholipids, and natural antioxidants such as rosmarinic acid, caffeic acid, quercetin, myricetin, and others have been reported in CSO [14]. CSO is thus largely recognized as one of the most valuable oils in the global market, with multifarious health benefits [15].

Realizing the significant nutritional properties and health-promoting medicinal values of CSO, its supplementation is now being increasingly recommended as an alternative and sustainable lipid source in aqua-feeds [16]. Recent studies directed towards the supplementation of chia seed powder in aqua-feeds of farmed Nile tilapia (*Oreochromis niloticus*) have revealed its beneficial influence on growth performance, haemato-immunological indices, stress biomarkers like glucose and cortisol, antioxidant enzymes, hepatic malonaldehyde, histopathological architecture, and disease resistance [17,18]. Another study conducted on CSO incorporation depicted its role in affecting growth, hematology, and the composition of fatty acids, as a partial or total replacement of fish oil in the diet of gilthead sea bream (*Sparus aurata*, L.) [19]. Further, a study related to the incorporation of chia seed powder in the diet of *O. niloticus* showed an increase in its nutritive value and antioxidant capacity [20]. Even with the expression of genes, the role of chia oil has been delineated in terms of the transcriptional regulation of metabolic genes associated with the biosynthesis of highly unsaturated fatty acids (HUFAs) [19]. Our recent study revealed that a diet supplemented with 1% CSO resulted in an augmentation of growth performance by positively influencing gut microbial composition and diversity in rohu fingerlings. The study also demonstrated that the group supplemented with 1.0% CSO showed an improvement in the maximum taxonomical richness of the major bacterial phyla, such as Verrucomicrobia, Actinobacteria, Fusobacteria, Bacteroidota, and Firmicutes, leading to enhanced intestinal immunity [21].

This study builds upon previous research on growth performance by investigating the effects of varying concentrations of CSO supplementation on hematological, immunological, and antioxidant parameters, as well as immune-responsive cytokine gene expression. To our knowledge, this represents the first comprehensive assessment of CSO as a plant-based alternative to fish oil in aquaculture, utilizing a combined biochemical and molecular approach. The findings are anticipated to provide valuable reference data for the aquaculture industry.

## 2. Materials and Methods

### 2.1. Formulation of Experimental Diets

Four different isoproteic and isoenergetic diets were prepared for the commencement of the feeding trial. Golden, bright-colored, 100% pure and natural, cold-pressed CSO was procured from the local market (Salvia cosmeceuticals, Pvt. Ltd.; New Delhi, India). The incorporation of CSO was carefully performed at graded levels of 0% (control), 1.0%, 2.0%, and 3.0% to ensure a total lipid percentage of 6.0%, in accordance with the nutritional requirements of *L. rohita* (Table 1). During diet formulation, the same ingredients were used, except for cod liver oil which was replaced with CSO. All the constituent ingredients in the individual diets were measured and mixed proportionately with distilled water to achieve a smooth dough, which was further autoclaved (Equitron^®^ Autoclave SLEFA) under reduced pressure (15 psi) for 20 min. Post sterilization, the dough was cooled to room temperature and vitamin–mineral premix was added. The formulated diets were finally recovered as 1 mm pellets through an automated pelletizer. The obtained pellets were dried at 60 °C for 36 h and then cooled at room temperature before being carefully bagged and stored in labeled airtight containers at −20 °C.

### 2.2. Culture Conditions and Design of the Experiment

The experiment in the present study was conducted at the School of Molecular Diagnostics, Prophylactics and Nanobiotechnology (SMDPN), ICAR-Indian Institute of Agricultural Biotechnology, Garhkhatanga, Ranchi. Live rohu fingerlings (n = 300) were transported aseptically to the fish housing facility after being procured from a commercial fish farm from the state fisheries department located in Doranda, Ranchi. Post transportation, the fish were subjected to prophylactic treatment with 1% salt solution followed by disinfection with 0.01% potassium permanganate (KMnO_4_) solution. The fingerlings were then transferred to a circular FRP tank (1000 L capacity) for acclimatization over a period of 15 days, during which they were fed twice with the commercial fish diet (containing 30% crude protein and 5% ether extract). Following acclimatization, 180 *L. rohita* fingerlings with an initial live weight of 19.74 ± 0.33 g were redistributed into 12 randomly assigned separate rectangular FRP tanks (300 L, capacity), with a stocking density of 15 fish per tank. The complete experimental set up comprised four different treatment groups in triplicates. All the rearing tanks were provided with the needed compressed aeration and were hand-fed their respective formulated diets for 60 days, twice a day at 10:00 am and 5:00 pm, daily, until visual satiety. The residual feed and the fecal matter were siphoned out after the last feeding of each day, along with an alternate day exchange of 20% of the culture water. Adjustments were made in the feeding rates every fortnight as per the change in the total biomass. Water quality parameters, namely water temperature (25.8–28.8 °C), pH (7.36–7.72), and dissolved oxygen (6.36–6.82 mg L^−1^), were maintained in their optimum range throughout the experimental period.

### 2.3. Fatty Acid Analysis of Experimental Diets

Fatty acid methyl esters were prepared from feed samples using saponification and esterification, following standard IUPAC methods. FAME analysis was performed using Thermo Scientific ITQ 900 Gas Chromatography-Ion Trap Mass Spectrometry (GC/IT-MS) (Waltham, MA, USA). A TR-FAME capillary column and MS (ITQ 900, Thermo Scientific) (Waltham, MA, USA) were used to quantify the fatty acids as per the standard methodology [22]. Helium was employed as the carrier gas. The ion source and transfer line temperatures were 220 °C and 250 °C, respectively. Constituents were identified and quantified using NIST Library, comparing the retention times and peak areas to those of standards (ME-14-KT and ME-19-KT, SUPELCO Analytical; Bellefonte, PA, USA); the individual constituents displayed by GC were calculated and are presented in Table 2.

### 2.4. Blood Sampling and Estimation of Haemato-Immunological Parameters

Blood collection was performed with 3 individual captured fish from each replicate tank, after being euthanized using clove oil (Merck, Darmstadt, Germany) (50 µL L^−1^). Blood was withdrawn by carefully rupturing the caudal vein with a 23-gauge needle of a 1.0 mL hypodermal syringe. To ensure proper blood collection, the syringes were pre-rinsed with EDTA anti-coagulant solution (2.7% *w*/*v*). The blood was then collected in both EDTA-coated heparinized tubes and non-heparinized tubes. The heparinized tubes were vigorously shaken to avoid the clotting of blood and kept at 4 °C. Conversely, non-heparinized tubes were stabilized in inclined position for 2 h at room temperature to ensure clot formation. Serum was subsequently isolated through centrifugation at 3500× *g* for 4 min at 4 °C. The isolated serum was then meticulously transferred to 1.5 mL Eppendorf tube and stored at −80 °C until needed.

Serum total protein was quantified using the Yumizen CR protein kit RTP0821(Horiba Medical, New Delhi, India), following the manufacturer’s instructions, with bovine serum albumin as the standard. The Erba Liquixx albumin kit 120233 (Transasia Bio-Medicals Ltd., Mumbai, India) was employed to determine the albumin content, following the manufacturer’s instructions. Globulin values were derived by subtracting albumin values from total protein values. Assays for serum glucose (RGLUL0821C), alanine aminotransferase (ALT) (RALTL0921), aspartate aminotransferase (AST) (RASTL0921), cholesterol (RCHOL0821), and triglyceride (RTRG0221) activities were conducted according to the manufacturer’s protocol, utilizing commercial kits (Yumizen CR, Horiba Medical, New Delhi, India). Serum C-reactive protein (CRP) was measured using the Erba T-LYX CRP kit 131959 (Transasia Bio-Medicals Ltd., Mumbai, India), as per the provided manual.

### 2.5. Evaluation of Anti-Oxidative and Protein Metabolic Enzymes

One fish from each replicate tank was aseptically dissected using sterile equipment to collect visceral tissue for the assessment of enzymatic assays. Tissue homogenates (5%) from the liver, kidney, and intestine were prepared using a tissue homogenizer (Omni Tissue Master Homogenizer, Kennesaw, GA, USA) with 0.25 M chilled sucrose and 1 mM EDTA solution. After centrifugation at 6000× *g* for 20 min at 4 °C, the supernatant was separated and stored at −20 °C. The estimation of protein in different tissue homogenates was carried out as per methodology [23], using the colorimetric assays performed employing UV-Vis spectrophotometer (Eppendorf Biospectrometer, AG22331, Hamburg, Germany). Superoxide dismutase (SOD) (EC 1.15.1.1) activity was determined based on the enzymatic oxidation of epinephrine to adrenochrome, following the standard method. A reaction mixture containing 50 μL tissue homogenate, 1.5 mL phosphate buffer, and 0.5 mL freshly prepared epinephrine was monitored for absorbance at 480 nm for 3 min [24]. Catalase (CAT) (EC 1.11.1.6) activity was measured using the standard method. A reaction mixture of 50 μL tissue homogenate, 2.45 mL phosphate buffer (50 mM; pH 7), and 1 mL hydrogen peroxide substrate solution was prepared, and the decrease in absorbance was recorded for 3 min at 240 nm [25]. Glutathione-S-transferase (GST) (EC 2.5.1.13) activity was determined according to the standard method, based on the formation of the adduct of 1-Chloro-2,4-dinitrobenzene (CDNB) and S-2, 4-dinitrophenyl glutathione, utilizing S-2,4-dinitrophenyl glutathione as the substrate. The activity was monitored by measuring the increase in absorbance at 340 nm against the blank [26].

AST (E.C.2.6.1.1) activity was assayed in the liver, kidney, and intestinal tissue homogenates as per the standard protocol. The substrate included 0.2 M of L-aspartic acid and 2 mM a-ketoglutarate in 0.05 M phosphate buffer (pH 7.4). The reaction was initiated by adding 0.1 mL of homogenate. Further, the mixture was incubated at 37 °C for 60 min and terminated by the addition of 0.5 mL of 1 mM 2,4-dinitrophenylhydrazine (DNPH) [27]. A similar procedure was adopted for ALT (E.C.2.6.1.2) enzyme activity, except the substrate comprised 0.2 M D alanine instead of aspartic acid.

### 2.6. RNA Isolation and Quantitative RT-PCR

After the assortment of serum and blood plasma samples, the same 3 fish from each replicate tank were aseptically dissected using sterile apparatus to obtain visceral tissue samples from the liver, head, kidney, and intestine. The collected tissue samples from each treatment group were pooled together and promptly stored at −20 °C in labeled 1.5 mL tubes containing RNA until further use for RNA extraction. Total cellular RNA was extracted from the tissues following the standard protocol provided by TAKARA BIO INC. The extracted RNA was resuspended in an appropriate amount of RNase-free water. The isolated RNA underwent treatment with RNase-free DNase I (Fermentas, MA, USA), followed by DNase I inactivation. RNA samples were quantified using a Nanodrop spectrophotometer (One^c^ Thermo scientific) (Waltham, MA, USA), and purity was assessed based on the A_260nm_/A_280nm_ ratio. The integrity of the intact RNA was visualized by electrophoresis on a 1% agarose gel containing 0.5 μg mL^−1^ ethidium bromide at 100 V in the Molecular Imager Gel Doc^TM^ XR+ Imaging System (Bio Rad, Hercules, CA, USA). The band intensity of 28S and 18S ribosomal RNA was assessed, and the tested RNA was then used for the expression analysis of various immune-responsive genes.

First-strand cDNA synthesis was performed using the TAKARA PrimeScript™ 1st strand cDNA Synthesis Kit in a thermocycler (Applied Biosystems ProFlex PCR System, Waltham, MA, USA), following the manufacturer’s instructions. Each reaction was conducted in a final volume of 20 μL buffer, consisting of 10 μL template RNA, 4 μL 5X PrimeScript Buffer, 1 μL Prime-Script RTase, 4 μL Primer Mixture, 0.5 μL RNase Inhibitor, and RNase-free water.

Quantitative real-time amplification of the prepared cDNA was conducted with Takara TB Green Premix Ex Taq (Tli RNaseH Plus, Shiga, Japan), using the StepOnePlus Real-time PCR system (Applied Biosystems, Waltham, MA, USA). Gene-specific primer pairs based on the rohu transcriptome data were employed, illustrated in Table 3. The reaction mixture, totaling 10 μL, comprised 1 μL of template cDNA, 0.5 μL each of primer pairs (5 pmole and 3 pmole for forward and reverse primers, respectively), 5 μL of TB Green Premix Ex Taq and 0.4 μL of nuclease-free water. The qPCR program involved pre-denaturation), denaturation (95 °C; 10 s), annealing (temperature refer Table 3; 15 s), and extension (72 °C; 20 s). A negative control without cDNA was included, while β-actin served as the reference house-keeping gene. Cytokine expression analysis for IL-1β, TNF-α, IFNγ, TLR22, and IL-10, based on 2^−ΔΔCt^ values was performed using the comparative threshold cycle calculation 2^−ΔΔCt^ method.

### 2.7. Statistical Analysis

Statistical analyses of the acquired data were conducted utilizing SPSS version 22 to assess significant variances between means. Each replicate tank was used as experimental unit. The standard error (SE) within the data was determined and presented as the mean ± standard error. To assess the normality and homogeneity of data variances, Shapiro–Wilk’s and Levene’s tests were employed. One-way Analysis of Variance (ANOVA) with confidence limits based on *p* ≤ 0.05 was employed among the treatment groups to compare significant differences. The values for relative fold change in the expression of cytokine genes were plotted using GraphPad Prism version 9.3.1 and further evaluated through comparisons at a 5% probability level.

## 3. Results

### 3.1. Haemato-Immunological Indices

Dietary supplementation of CSO in rohu fingerlings resulted in a significant increase (*p* < 0.05) in total protein and globulin levels at the 1% inclusion level compared to the control and 3% CSO-supplemented group. However, the level of albumin as well as the A/G ratio significantly declined in the CSO (1) group in comparison to the control (Table 4). The lowest total protein and globulin levels were observed in the 3% CSO-supplemented group, which was similar to the control group. The A/G ratio under the maximum supplementation level of CSO was found to be the highest compared to other CSO-supplemented groups. CSO supplementation also led to a decrease in serum glucose levels, with the highest (*p* < 0.05) decline observed in the group with 3% CSO. Cholesterol levels showed a decreasing trend in *L. rohita* fingerlings fed with either 1% or 2% CSO supplementation. A significant decline (*p* < 0.05) in triglyceride levels was observed in the CSO (1) group, followed by the CSO (2) group. The 1% CSO-fed group also exhibited the lowest (*p* < 0.05) level of CRP compared to other treatment groups (Table 4).

### 3.2. Enzyme Assays

Table 5 illustrates the levels of antioxidative stress enzymes, specifically catalase, SOD, and GST, in the liver, kidney, and intestine of the rohu fingerlings after 60 days of dietary supplementation with CSO. CSO incorporation affected catalase activity in different organs. The 1% and 2% CSO-fed groups witnessed a significant (*p* < 0.05) increase in catalase activity in the intestine. Higher liver catalase activity was observed CSO (1), while the maximum catalase level in the kidney was detected in CSO (2).

SOD activity was higher in the 1% CSO-fed group in all tested organs, with the kidney showing the highest (*p* < 0.05) activity in the 1% CSO group. GST activity also showed a noticeable (*p* < 0.05) enhancement in the liver of rohu fed with 1% CSO supplementation.

Table 6 represents the AST and ALT activity in the liver, kidney, and intestine of fingerlings fed with CSO-supplemented diets. Fish fed 1% and 2% CSO had significantly (*p* < 0.05) higher hepatic AST activity than the other treatment groups. AST activity in the intestine demonstrated the highest value in the 1% CSO group, but it was comparable with other treatment groups except the highest-supplemented CSO (3) group. CSO supplementation also affected ALT activity in all tested organs, with the liver and kidney exhibiting the highest activity (*p* < 0.05) in the 1% CSO group compared to other treatments.

### 3.3. Expression Analysis of Immune-Responsive Cytokine Genes

**Figure 1:**
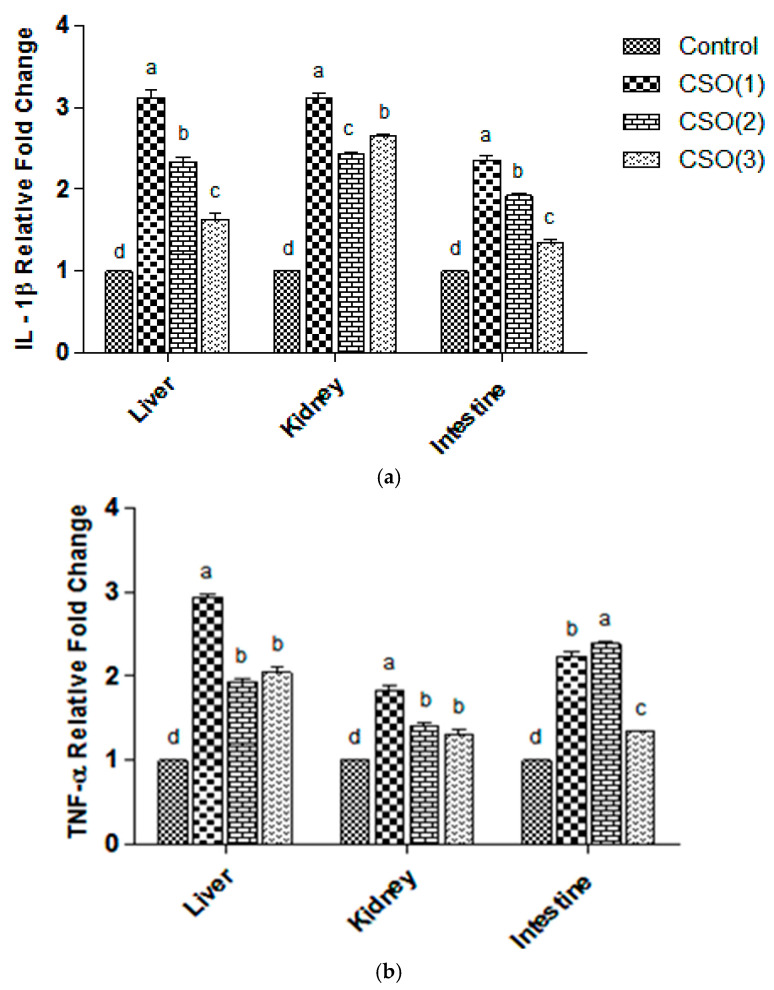

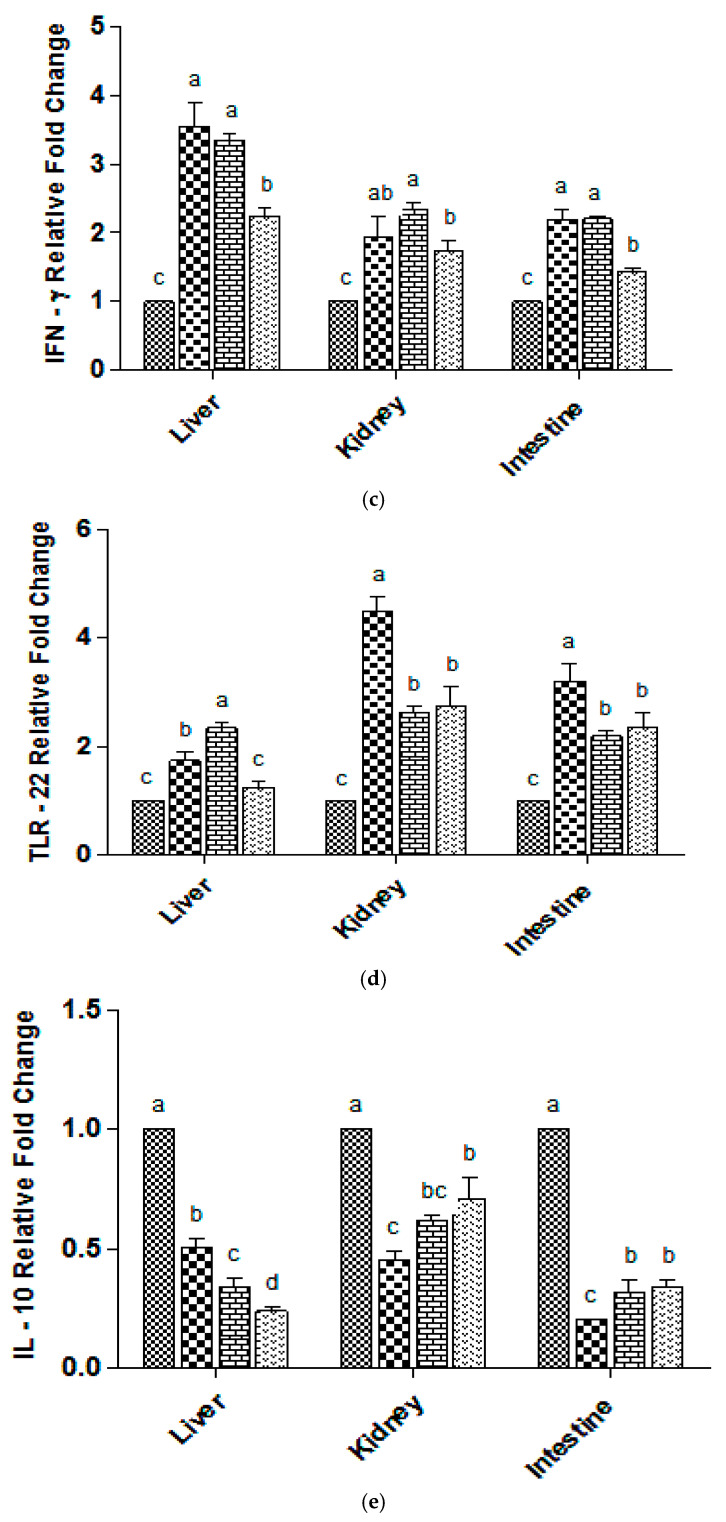
Expression of IL-1β gene (**a**), TNF-α (**b**), IFN-γ (**c**), TLR22 (**d**), and IL-10 (**e**) in the liver, kidney, and intestine relative to β-actin after 60 days of dietary trial with CSO supplementation in *Labeo rohita* fingerlings. Statistically significant modulation in the expression of genes relative to the control group and CSO-supplemented dietary groups is displayed. Different superscripts (a, b, c, and d) on the uppermost part of error bars show significant differences (*p* < 0.05). Values are represented as mean ± SE, n = 3.

Thes study evaluated the mRNA expression of five important immune-regulatory cytokine genes in the liver, kidney, and intestine of rohu fingerlings supplemented with CSO (Figure 1a–e). Noteworthy variations in the expression of IL-1β, TNF-α, IFN-γ, TLR22, and IL-10 were observed. All the groups recorded upregulated levels of the pro-inflammatory cytokines as well as a downregulation of the anti-inflammatory cytokine IL-10.

IL-1β expression showed substantial variation, with the highest upregulation in the 1% CSO group in the liver, kidney, and intestine. A significant decline in IL-1β expression relative to the CSO (1) group was also noted at higher doses of 2% and 3% CSO. TNF-α expression exhibited modulation patterns similar to IL-1β, with the highest (*p* < 0.05) elevated expression at 1% CSO supplementation in the liver and kidney. Among CSO-supplemented groups, intestine cells exhibited the lowest expression of IL-1β, TNF-α, and IFN-γ in the 3% CSO-supplemented group. IFN-γ expression was significantly upregulated in the liver and intestine with 1% and 2% CSO supplementation. All the tested organs exhibited comparable upregulation of IFN-γ at both 1% and 2% CSO. TLR22 expression showed the highest (*p* < 0.05) upsurge in the 1% CSO group in the kidney and intestine, while the liver recorded the highest (*p* < 0.05) upregulation at 2% CSO supplementation.

Contrary to the pro-inflammatory cytokine genes, IL-10, an anti-inflammatory gene, exhibited downregulated expression in all tissues. The intestinal cells exhibited a significant down-regulation of IL-10 expression in the 1% CSO group, whereas the liver demonstrated the highest downregulation in the 3% CSO group (*p* < 0.05).

## 4. Discussion

The stagnating raw material for fish oil production in the formulation of aquafeeds has demarcated a dire need for alternative lipid sources as replacements [31]. Plant-originated vegetable oils have emerged as the chief dietary substitutes to offset the pressure on wild marine fish stock and promote favorable immune nutrition in fish fed with the corresponding formulated diets [8]. Many studies in the past have demonstrated remarkable therapeutic potential and health benefits of CSO in animal feeds [32]. Our recent research findings on 16S rRNA amplicon sequencing indicate that rohu fed 1% CSO showed a positive change in microbial diversity and composition, and exhibited an increased relative abundance of bacterial communities including Verrucomicrobia, Actinobacteria, Fusobacteria, Bacteroidota, and Firmicutes. The same study also depicted significant improvements in weight gain (WG) %, specific growth rate (SGR), and feed conversion ratio (FCR) in the group supplemented with the lowest level of 1% CSO [21].

Investigation into the impact of CSO inclusion in the practical diets of fish has gained significant momentum from health and immune perspectives [7]. Therefore, the present investigation is an extension of the work aligned with our previous finding on growth performance validated through various physiological, biochemical, and immunological endpoints.

Haemato-immunological indices serve as powerful insights into the nutritional, physiological, and clinical status of a host, demarcated by alterations in feed and feeding regime [33]. The present observations of significantly enhanced total protein and globulin levels could be linked to improved immune properties or stronger immunity associated with the increased levels of these non-specific immune variables [34]. Similar results related to the haemato-immunological indices were observed during the supplementation of chia seed powder in Nile tilapia fingerlings at 10–15 g/kg feeding rate [17]. Elevation in total protein levels in the serum could be correlated to improvement in hepatic tissue function by the potent action of essential oils including CSO [35]. In addition, the above study also reported a reduction in serum triglycerides and cholesterol levels with the supplementation of chia seed powder in Nile tilapia fingerlings, similar to the outcome obtained in the present study [35]. Equivalent results depicting the modulation of serum lipid profile upon the administration of chia seed powder were also exhibited in Nile tilapia exposed to low-temperature stress [18]. This effect can be associated with the occurrence of PUFAs and phytosterols in chia seed oil, which probably influence the expression of genes governing the metabolism of lipids [36]. This can also be related to the reduction in low-density lipoprotein (LDL) cholesterol and triglycerides mediated by the action of omega-3 fatty acid present in the endosperm of the chia seeds [37]. The biological activity of CSO has been further confirmed by various studies on animal models. A study in this regard advocated the anti-diabetic and hypoglycemic effects of chia oil upon its replacement of soybean oil in high-fat, fructose-rich diets fed to Wistar rats [38]. Other studies have reported the hypolipidemic and fat-reducing effects of chia oil in terms of non-esterified fatty acids, triglycerides (TGs) and total cholesterol (TC) in rats [39,40]. Replacement of corn oil with chia oil also resulted in similar output [39], wherein the supplementation as a lipid source restored hypolipidemic or normal conditions.

Serum glucose is commonly considered to be a stress indicator that depicts hormone-responsive changes to variations in diets. Previous studies have shown variable results relative to our findings. For instance, results in a similar line to the present study have been obtained as an impact of rosemary oil in young great sturgeon (Huso huso) at a similar supplementation level of 1.0% [41]. These lowered glucose values are often indicative of an improved antioxidant defense system and increased insulin production in the pancreas [41]. However, no significant changes in the level of glucose were observed upon the dietary replacement of 60% fish oil with chia oil in gilthead sea bream, (*Sparus aurata*) [19], which may be due to the ability of the cells to maintain glucose homeostasis upon the partial replacement of fish oil. However, promising results of chia seed powder (CSP) in Nile tilapia have been demonstrated in a relatively recent study, wherein lowered glucose values were observed when supplemented with 4.5 and 6 g/kg CSP [18]. This glucose-lowering ability of chia seed components, both powder and oil, may be correlated to the presence of natural antioxidants such as chlorogenic acid and caffeic acid as well as flavonoids, namely myricetin, kaempferol, and quercetin, in chia seed [42]. Moreover, these polyphenols act as potent scavengers of free radicals, thus alleviating oxidative stress [43,44], as evident in our results on antioxidative marker enzymes.

C-reactive protein is an important inflammatory marker. Currently, to the best of our knowledge, there are no parallel reports available pertaining to the impact of chia seed oil on the serum CRP in any fish species. However, the effects of chia seed or its derivatives have been observed in other animal models like rats and rabbits. The highly significant reduction in the levels of CRP observed in the present study aligns with the observation of supplementation of CSO in obese rats [45]. This improvement may be attributed to the presence of phenolic compounds, mucilage, phytosterols, and omega-3 fatty acids in CSO. Declining levels of CRP have also been observed after the supplementation of tea tree oil or lemongrass essential oil in broilers [46].

The incorporation of CSO, similarly to any other supplemented nutrient or feed additive, tends to influence the activation and transcription of genes involved in enzyme activities [40]. The current research also witnessed a significant stimulation of the endogenous antioxidant enzyme system comprising SOD, catalase, and GST. These enzymes play a crucial role in conserving redox homeostasis [47]. Cumulative stimulation of both the pioneer antioxidative enzymes, SOD and catalase, was observed with the supplementation of *Z. multiflora* extract in *O. mykiss* [48]. Another study substantiating our results demonstrated a significant modulation of the above-mentioned antioxidant enzymes in Nile tilapia fingerlings upon supplementation with chia seed powder at a level of 10 g/kg feed [17], which is equivalent to the supplementation level of 1.0% achieved in our study. Similar results were also portrayed when chia seed powder was supplemented at even lower rates of 4.5 and 6 g/kg in Nile tilapia fingerlings [18]. These results highlight the potent antioxidant activity of both the chia seed components, namely chia seed powder and CSO. These properties are further attributed to the abundance of phenolic acids such as caffeic acid, cinnamic acid, chlorogenic acid, ferulic acid, gallic acid, and p-coumaric, acid; flavonoids such as lignans, quercetin, rutin, kaempferol, epicatechin, and apigenin; and tocopherols, responsible for the absorption and neutralization of free radicals as well as the decomposition of peroxides [49,50]. In addition, the hepatic antioxidative biomarkers, viz. SOD and catalase activities, were also significantly increased upon supplementation of oregano oil in common carp (*Cyprinus carpio* L.), which is in agreement with our results [51]. The increase in the activities of marker antioxidant enzymes has also been noticed, after CSO supplementation in obese rats [38]. The study suggested that dietary CSO exerted a hypolipidemic effect and enhanced the activities of antioxidant enzymes, which contributed to the attenuation of lipid peroxidation in the rats [38].

In addition, the evaluation of serum activities of key protein metabolic enzymes, namely ALT and AST, was also undertaken in this study. These liver biomarker enzymes are related to the maintenance of the health and physiological status of the host as major components of nutrient metabolism [52]. A study substantiating our findings displayed higher levels of ALT and AST enzymes upon administration of menthol essential oil in *O. niloticus* [53]. Results in support of the current study have also been reported with increasing dietary levels of palm oil in the large yellow croaker (*L. crocea*) as well as *O. niloticus* [54,55]. The similarity in results suggests the alleviating capacity of the antiradical components of the supplemented essential oils against oxidative stress.

Gene expression profiles along with other key immunological indices provide a holistic overview of diet-induced changes in fish health and immunity [7]. Thus, the expression pattern of different immune-responsive cytokine genes was analyzed in the current study with respect to the inclusion of CSO in the diets of L. rohita fingerlings. The tissues used for the evaluation of the effects of CSO on fish health were selected mainly based on their role in immunomodulation. TNF-α and IL-1β belong to the category of early-expressed pro-inflammatory cytokines, which play a crucial role in orchestrating an inflammatory response by stimulating immune cells to undergo phagocytosis. IFN-γ is another important category of the regulatory pro-inflammatory cytokines, acting towards enhanced immune response [56].

Studies corroborating our results pertaining to the upregulation of pro-inflammatory cytokines in different immune organs have been advocated in the past. For instance, a related expression profile showing substantial elevation in the level of immune responsive genes, IL-1β and TNFα, has been exhibited in the intestinal cells of turbot (*Scophthalmus maximus*) upon feeding oregano oil at a rate of 1 mL/kg [57]. Significantly upregulated expression patterns have also been achieved for the genes in both the head and kidney of rainbow trout (*Oncorhynchus mykiss*) juveniles supplemented with 2 g/kg of Zataria multiflora extract (thyme), mainly attributed to the high concentration of thymol and carvacrol essential oil [48]. In addition, an increased IL-1β transcript level was presented at 15–20 g/kg supplementation of oregano essential oil in common carp [51]. On the contrary, the level of TNFα was found to decrease significantly in koi carp (C. carpio) fed with 500 mg/kg of oregano essential oil. Nevertheless, the study did not reveal any significant disparity in IL-1β levels. The difference could be associated with the corresponding alteration in the gut microbiota, particularly the decrease in Vibrio species, of koi carp upon supplementation with oregano oil, contributing to the anti-inflammatory response [58]. Similarly, an increased level of IFNγ, corroborating our results, was found in the liver of the large yellow croaker (*Larimichthys crocea*) upon the replacement of fish oil with dietary palm oil [55]. Interestingly, higher transcript levels of IFNγ were also achieved in the study with menthol essential oil on Nile tilapia [53]. The elevated levels of the immune-related genes in Nile tilapia could be attributed to the stimulation of T lymphocytes towards cytokine secretion [59].

Next, in agreement to our results, an increase in the level of TLR22 was also noted by [55] in the liver of large yellow croaker fed with dietary palm oil. A similar increase was witnessed upon the inclusion of rapeseed oil and peanut oil within the diets of hybrid grouper (*Epinephelus fuscoguttatus* ♀ × *E. lanceolatus* ♂). The study correlated the effect to the activation of TLR-NF-κB signaling pathway by dietary fatty acids in the peanut oil-supplemented group [60]. This pathway was also reported to account for the consequent downregulation of the anti-inflammatory cytokine IL-10 in palm-oil supplemented large yellow croaker, which further substantiates our finding [55]. On the other hand, a contradicting upregulation of IL-10 in the liver was simultaneously expressed in oregano oil-supplemented *C. carpio* [51]. This upregulation disagrees with the corresponding downregulation of the same gene in the present study. The differences might have arisen due to varying fish species as well as the varying inclusion forms of lipid sources at differential supplementation or replacement rates.

## 5. Conclusions

Thus, it can be summarized that the supplementation of CSO significantly improved the non-specific immunity in *L. rohita* fingerlings. The supplemented diets exhibited markedly enhanced haemato-immunological indices and metabolic enzyme levels as well as favorably affected expression patterns of key immune-responsive genes in targeted organs of *L. rohita*. Among the different graded levels, the minimum supplementation level of 1.0% proved to be the optimum inclusion dosage, characterized by the highest response on the evaluated antioxidant and immune biomarkers. CSO at 1.0% supplementation can thus be hypothesized as a prospective dietary intervention strategy to act as a sustainable vegan substitute to fish oil in rohu aquaculture and therefore endorses the sustainable expansion of the blue economy.

## Figures and Tables

**Table 1:** Composition of the experimental diets (g kg^−1^ on dry matter basis) fed to *L. rohita* fingerlings during the experimental period of 60 days.

Ingredients	Control	CSO (1)	CSO (2)	CSO (3)
Soybean meal ^a^	310	310	310	310
Fish meal ^a^	80	80	80	80
Groundnut meal ^a^	170	170	170	170
Wheat flour ^a^	240	240	240	240
Corn flour ^a^	110	110	110	110
Cod liver oil ^a^	60	50	40	30
Chia seed oil ^a^	00	10	20	30
Vitamin + mineral mix ^b^*	20	20	20	20
BHT ^d^	05	05	05	05
Vitamin C ^c^	05	05	05	05
Total	1000	1000	1000	1000
Proximate analysis (g kg^−1^)				
Crude protein	352.3	351.4	350.8	349.8
Crude lipid	71.3	71.4	71.6	71.5
Ash	96.1	95.7	95.5	95.8

All the quantities given in the table are measured in grams. ^a^ Procured from a local market. ^b^ Prepared manually and all components from Himedia Ltd. ^c^ SD Fine Chemicals Ltd., Mumbai, India. ^d^ Himedia laboratories, Mumbai, India. ^b^* Composition of vitamin mineral mix (quantity/250 g starch powder): vitamin A 550,000 IU; vitamin D3 110,000 IU; vitamin B1 20 mg; vitamin B2 200 mg; vitamin E 75 mg; vitamin K 100 mg; vitamin B12 0.6 μg; calcium pantothenate 250 mg; nicotinamide 1000 mg; pyridoxine 100 mg; Mn 2700 mg; I 100 mg; Fe 750 mg; Cu 200 mg; Co 45 mg; Ca 50 g; P 30 g. CSO—chia seed oil.

**Table 2:** Fatty acid profile (% total fatty acids) of experimental diets.

Fatty Acids (%)	Control	CSO (1)	CSO (2)	CSO (3)
C12:0	1.15	1.45	1.8	2.08
C14:0	5.86	4.34	3.64	2.72
C16:0	16.63	16.26	15.04	14.81
C17:0	3.22	3.16	2.59	2.11
C18:0	3.42	3.51	3.65	3.83
C16:1	5.73	5.03	4.33	2.71
C18:1	11.72	12.80	15.73	17.38
C20:1 n-9	2.94	3.01	3.37	3.73
C22:1 n-9	3.03	3.45	4.23	4.59
C18:2 n-6 (LA)	5.86	7.93	10.49	12.65
C18:3 n-3 (ALA)	2.47	6.49	13.46	19.87
C20:3 n-3	1.08	0.94	0.71	0.51
C20:5 n-3 (EPA)	7.61	6.09	4.33	2.69
C22:6 n-3 (DHA)	10.84	8.56	6.43	3.19
∑ SFA	30.28	28.72	26.72	25.55
∑ MUFA	23.42	24.29	27.66	28.41
∑ n-3 PUFA	22.01	22.08	24.93	26.26
EPA + DHA	18.45	14.65	10.76	5.88
EPA/DHA ratio	0.70	0.71	0.67	0.84

CSO—chia seed oil; SFA—saturated fatty acid; MUFA—monounsaturated fatty acid; PUFA—polyunsaturated fatty acids; LA—linoleic acid; ALA—alpha linolenic acid; EPA—eicosapentaenoic acid; DHA—docosahexaenoic acid; EPA + DHA—(C20:5 n-3 + C22:6 n-3).

**Table 3:** Gene-specific primers, annealing temperature, and accession number used for qPCR analysis in the study.

Gene	Primer Sequence	Annealing Temp.	Accession Number/Reference
TNF-α	Forward-5′CCAGGCTTTCACTTCAGG3′ Reverse-5′GCCATAGGA ATCGGAGTAG3′	51.6 °C	FN543477
IL-10	Forward-5′GACATCAAAGAGAGTCAAGCACTTATAGT3′ Reverse-5′TGCAGAGTATTCAGATTTGACTCAAGTC3′	61.5 °C	HM228928
IL1-β	Forward-5′ATCTTGGAGAATGTGATCGAAGAG3′ Reverse5′GATACGTTTTTGATCCTCAAGTGTGAAG3′	57.5 °C	AM932525
TLR-22	Forward-5′TCACCCCATTTCGAGGCTAACAT 3′ Reverse-5′CGGAGGTAGGTTCGTTTCTTCA 3′	51.6 °C	[28]
IFN-γ	Forward-5′TGTGTTCCTCAACAGACACC 3′ Reverse-5′TGGAGAAACAGTTGACTCATGTG 3′	61.5 °C	[29]
β-actin	Forward-5′GACTTCGAGCAG GAGATGG3′ Reverse-5′CAAGAAGGATGGCTGGAACA3′	55.3 °C	[30]

**Table 4:** Hematological parameters of *L. rohita* fingerlings fed with various chia seed oil (CSO)-supplemented diets for 60 days.

Treatments	Total Protein (g dL^−1^)	Albumin (g dL^−1^)	Globulin (g dL^−1^)	A/G Ratio	Glucose (mmol L^−1^)	Cholesterol (mmol L^−1^)	Triglyceride (mmol L^−1^)	CRP (mg L^−1^)
Control	1.38 ^bc^ ± 0.01	0.42 ^a^ ± 0.03	0.96 ^b^ ± 0.01	0.43 ^a^ ± 0.01	12.89 ^a^ ± 0.33	3.20 ^b^ ± 0.06	2.87 ^a^ ± 0.04	96.67 ^a^ ±1.66
CSO (1)	1.58 ^a^ ± 0.03	0.28 ^c^ ± 0.01	1.15 ^a^ ± 0.03	0.24 ^b^ ± 0.01	11.72 ^ab^ ± 0.63	2.81 ^c^ ± 0.06	2.39 ^c^ ± 0.03	66.98 ^d^ ± 0.89
CSO (2)	1.41 ^b^ ± 0.01	0.29 ^c^ ± 0.03	1.11 ^a^ ± 0.01	0.26 ^b^ ± 0.02	11.28 ^bc^ ± 0.26	2.99 ^c^ ± 0.05	2.57 ^b^ ± 0.03	77.51 ^c^ ± 2.62
CSO (3)	1.34 ^c^ ± 0.02	0.40 ^b^ ± 0.01	0.94 ^b^ ± 0.02	0.42 ^a^ ± 0.03	10.38 ^c^ ± 0.20	3.50 ^a^ ± 0.03	3.01 ^a^ ± 0.07	89.05 ^b^ ± 0.89

Different superscripts (a, b, c, d) in same column represent significant differences (*p* < 0.05). Data expressed as mean ± SE, (n = 3); CRP—C-reactive protein; CSO—chia seed oil; A/G—albumin/globulin ratio.

**Table 5:** Catalase, SOD, and GST activities of *Labeo rohita* fed with different levels of dietary chia seed oil (CSO) for 60 days.

	Anti-Oxidative Assays								
Treatments	CAT Liver	CAT Kidney	CAT Intestine	SOD Liver	SOD Kidney	SOD Intestine	GST Liver	GST Kidney	GST Intestine
Control	19.57 ^ab^ ± 1.18	21.84 ^b^ ± 0.76	14.11 ^b^ ± 0.86	48.55 ^ab^ ± 2.31	31.43 ^c^ ± 1.03	23.35 ± 1.28	6.61 ^b^ ± 0.57	4.68 ^ab^ ± 0.09	2.14 ± 0.15
CSO (1)	22.54 ^a^ ± 0.60	23.51 ^ab^ ± 0.65	18.44 ^a^ ± 1.12	56.46 ^a^ ± 2.16	41.54 ^a^ ± 1.93	27.18 ± 1.89	9.32 ^a^ ± 0.36	5.58 ^a^ ± 0.64	2.91 ± 0.41
CSO (2)	20.88 ^ab^ ± 1.14	25.53 ^a^ ± 0.75	17.93 ^a^ ± 0.27	50.37 ^ab^ ± 1.83	37.46 ^b^ ± 2.41	27.21 ± 1.12	7.18 ^b^ ± 0.42	5.32 ^ab^ ± 0.50	2.69 ± 0.19
CSO (3)	19.28 ^b^ ± 0.57	22.33 ^b^ ± 0.61	13.48 ^b^ ± 0.58	46.98 ^b^ ± 3.08	30.77 ^c^ ± 1.79	24.55 ± 0.99	6.93 ^b^ ± 0.74	3.86 ^b^ ± 0.27	2.36 ± 0.29

Values in the same column with different superscripts (a, b, c) differ significantly (*p* < 0.05). Data are expressed as mean ± SE, (n = 3); CAT—catalase; SOD—superoxide dismutase; GST—glutathione-s-transferase, units mg protein^−1^.

**Table 6:** Aspartate aminotransferase (AST) and alanine aminotransferase (ALT) activity in tissues of *L. rohita* fingerlings fed with chia seed oil (CSO)-supplemented diets for 60 days.

Treatments	AST			ALT		
	Liver	Kidney	Intestine	Liver	Kidney	Intestine
Control	11.28 ^b^ ± 0.57	11.08 ± 1.09	14.86 ^ab^ ± 1.02	15.34 ^bc^ ± 0.55	12.66 ^b^ ± 0.45	9.29 ^c^ ± 0.45
CSO (1)	14.42 ^a^ ± 0.65	12.22 ± 1.00	16.52 ^a^ ± 0.43	18.61 ^a^ ± 0.58	15.21 ^a^ ± 0.17	11.07 ^b^ ± 0.73
CSO (2)	15.92 ^a^ ± 0.76	10.17 ± 0.16	14.92 ^ab^ ± 0.54	16.78 ^b^ ± 0.39	13.69 ^b^ ± 0.56	13.04 ^a^ ± 0.41
CSO (3)	11.37 ^b^ ± 0.49	10.16 ± 0.84	12.73 ^b^ ± 0.46	14.03 ^c^ ± 0.15	12.36 ^b^ ± 0.34	10.37 ^bc^ ± 0.28

Values in the same column with different superscripts (a, b, c) differ significantly (*p* < 0.05). Data are expressed as mean ± SE, (n = 3); ALT: nmole of sodium pyruvate formed/mg protein/min at 37 °C, AST: nmole oxaloacetate released/min/mg protein at 37 °C.

## Data Availability

The datasets generated during and/or analyzed during the current study will be made available by the corresponding author on reasonable request.
